# State-level population estimates of sexual minority adolescents in the United States: A predictive modeling study

**DOI:** 10.1371/journal.pone.0304175

**Published:** 2024-06-27

**Authors:** Johannes O. Ferstad, Maria Aslam, Li Yan Wang, Katherine Henaghan, Jiayi Zhao, Jingjing Li, Joshua A. Salomon

**Affiliations:** 1 Department of Management Science and Engineering, Stanford University School of Engineering, Stanford, California, United States of America; 2 Office of the Director, National Center for HIV, Viral Hepatitis, STD, and TB Prevention, Centers for Disease Control and Prevention, Atlanta, United States of America; 3 Division of Adolescent and School Health, National Center for HIV, Viral Hepatitis, STD, and TB Prevention, Centers for Disease Control and Prevention, Atlanta, United States of America; 4 Department of Health Policy, School of Medicine, Stanford University, Stanford, California, United States of America; University of Maribor, SLOVENIA

## Abstract

**Purpose:**

The Youth Risk Behavior Survey (YRBS) among high school students includes standard questions about sexual identity and sex of sexual contacts, but these questions are not consistently included in every state that conducts the survey. This study aimed to develop and apply a method to predict state-level proportions of high school students identifying as lesbian, gay, or bisexual (LGB) or reporting any same-sex sexual contacts in those states that did not include these questions in their 2017 YRBS.

**Methods:**

We used state-level high school YRBS data from 2013, 2015, and 2017. We defined two primary outcomes relating to self-reported LGB identity and reported same-sex sexual contacts. We developed machine learning models to predict the two outcomes based on other YRBS variables, and comparing different modeling approaches. We used a leave-one-out cross-validation approach and report results from best-performing models.

**Results:**

Modern ensemble models outperformed traditional linear models at predicting state-level proportions for the two outcomes, and we identified prediction methods that performed well across different years and prediction tasks. Predicted proportions of respondents reporting LGB identity in states that did not include direct measurement ranged between 9.4% and 12.9%. Predicted proportions of respondents reporting any same-sex contacts, where not directly observed, ranged between 7.0% and 10.4%.

**Conclusion:**

Comparable population estimates of sexual minority adolescents can raise awareness among state policy makers and the public about what proportion of youth may be exposed to disparate health risks and outcomes associated with sexual minority status. This information can help decision makers in public health and education agencies design, implement and evaluate community and school interventions to improve the health of LGB youth.

## Introduction

Population-based data on adolescent health behaviors and experiences are essential for planning and evaluating health promotion programs, tracking progress toward health goals, and understanding the effectiveness of interventions to mitigate health risk behaviors [1]. An important objective in the monitoring of adolescent health is to understand and reduce health disparities [2]. Sexual minority youth, including those identifying as lesbian, gay, or bisexual (LGB) and those reporting having same-sex partners, face stigma and discrimination that can place them at higher risks for negative health outcomes [3–5]. For example, compared to their heterosexual peers, LGB youth are more likely to report having felt sad or hopeless, been bullied at school, forced to have sex, used illicit drugs, misused prescription opioids, or seriously considered suicide [6, 7]. Studies have shown that LGB youth are more likely than their heterosexual peers to report a range of sexual risks, such as having sexual intercourse before age 13, having multiple sexual partners, or having sex without a condom [2, 6, 8, 9]. These experiences can in turn increase risks of mental health problems, human immunodeficiency virus (HIV) infection, other sexually transmitted diseases, and pregnancy. For examples, young gay and bisexual males have disproportionately high rates of HIV (69% of all new HIV diagnoses in 2019 were among gay and bisexual men, and those aged 13–34 accounted for 65% of all cases among gay and bisexual men), syphilis, and other sexually transmitted infections (STI) [10, 11]; adolescent bisexual females are more likely to have ever been pregnant than their heterosexual peers [12, 13].

Estimation of the population size of LGB youth is critical for understanding the scope of disparities at a population level and developing effective interventions to address the overall health needs of the LGB population. For example, population estimates of adolescent sexual minority males can not only help public health practitioners determine the burden of HIV and other STI in this population, but also help guide public health policies, programmatic efforts, and resources to effectively prevent and mitigate these infection risks, such as allowing minors to consent to pre-exposure prophylaxis services, educating health care providers about service delivery, and promoting inclusive sexual health education and gay-straight alliances in schools.

The Youth Risk Behavior Surveillance System, which includes surveys among high school students in most US states, includes standard questions about sexual identity and sex of sexual contacts, but these questions have not been included in every state survey [2]. To address the information gaps resulting from omission of these questions from some surveys, we developed a new predictive model using machine learning methods and state Youth Risk Behavior Survey (YRBS) data. We predicted survey responses to questions about LGB identity and having sex with any same-sex partners in order to produce aggregate-level estimates for states that do not have this information directly available from surveys.

## Materials and methods

### Data

We used state high school YRBS data from 2013, 2015, and 2017 [2, 6, 14]. These surveys are conducted by state education and health agencies, designed in reference to a standard set of questions, with allowance for state agencies to add or delete questions depending on their programmatic or policy needs, following guidance from the Centers for Disease Control and Prevention (CDC). We obtained state-level datasets either from CDC or directly from individual states. We only included those surveys that provided weighted data to produce representative samples of the high school students in each state. Across all 50 states, three (Minnesota, Oregon, and Washington) did not conduct a YRBS in any of the three included study years. Table 1 and S1 Table provide summaries of the data used in our analysis. The full list of survey questions and variables included in the dataset can be found in the YRBS Combined Datasets User’s Guide [15].

**Table 1:** Data used to predict proportions of students in grades 9–12 reporting lesbian, gay, or bisexual identity and proportions reporting any same-sex sexual contacts in the United States in 2017.

	Lesbian, gay, or bisexual identity		Any same-sex sexual contact	
Statistic	Outcome available (training data)	Outcome unavailable (prediction data)	Outcome available (training data)	Outcome unavailable (prediction data)
Number of respondents included	382,251	35,545	320,410	92,406
Number of states included with any YRBS data	32	17	30	21
Number of states included with 2017 YRBS data	30	9	26	13
Number of states included with 2015 YRBS data	25	5	23	5
Number of states included with 2013 YRBS data	12	3	12	3
Number of states also asking the other focal question in 2017 YRBS	26	0	26	4
Unique years of YRBS data included per state, number, mean [min, max]	2.1 [1, 3]	1 [1, 1]	2.0 [1, 3]	1 [1, 1]
Number of questions included per state, median [min, max]	79 [30, 93]	79 [20, 90]	81 [30, 93]	75 [20, 90]
Observed proportion by state per 100k population, mean (SD)	9,375 (1,228)	-	7,079 (957)	-

S1 Table contains a detailed data summary for each state. We only used the most recent year of data from each state to predict 2017 proportions.

Abbreviations: LGB, lesbian, gay, or bisexual; YRBS, Youth Risk Behavior Survey; SD, standard deviation

### Measures

We defined two primary outcome variables corresponding to YRBS questionnaire items as follows:

#### Self-reported LGB identity

This outcome was coded based on individual responses to Q67 in the 2017 Combined YRBS Dataset: *Which of the following best describes you*? *A*. *Heterosexual (straight) B*. *Gay or lesbian C*. *Bisexual D*. *Not sure*. We coded the binary minority sexual identity outcome (“LGB Identity”) as 1 if the recorded answer was “Gay or Lesbian” or “Bisexual”. All other responses, including missing responses, were coded as 0.

#### Reported (any) same-sex sexual contact

This outcome was coded based on individual responses to the *sex* question and Q66 in the 2017 Combined YRBS Dataset. The *sex* question asks *What is your sex*? *A*. *Female B*. *Male*. Q66 asks *During your life*, *with whom have you had sexual contact*? *A*. *I have never had sexual contact B*. *Females C*. *Males D*. *Females and males*. The following respondents were assigned a 1 for the binary sexual contacts outcome (“Any Same-Sex Contact”): (1) respondents answering “Females and males” to Q66, (2) respondents responding “Male” to the *sex* question and “Males” to Q66, and (3) respondents responding “Female” to the *sex* question and “Females” to Q66. All other responses, including missing responses, were coded as a 0.

Our main analyses focused on predictions for the two primary outcomes for both sexes combined. Because some public health risks and programs vary for adolescent males and females–for example, adolescent sexual minority males are identified as a population with elevated risk of acquiring HIV and other STI–we also produced sex-stratified predictions of proportions reporting any same-sex sexual contacts (see S2 Table for sex-stratified data summary). Independent variables for prediction included responses to all other survey questions in YRBS. Missing responses on the independent variables were replaced with the modal response in the full dataset (across all the included states), and an additional covariate was created for each question comprising an indicator for whether the response was missing for a particular respondent.

### Analysis

#### Overview

**Fig 1:**
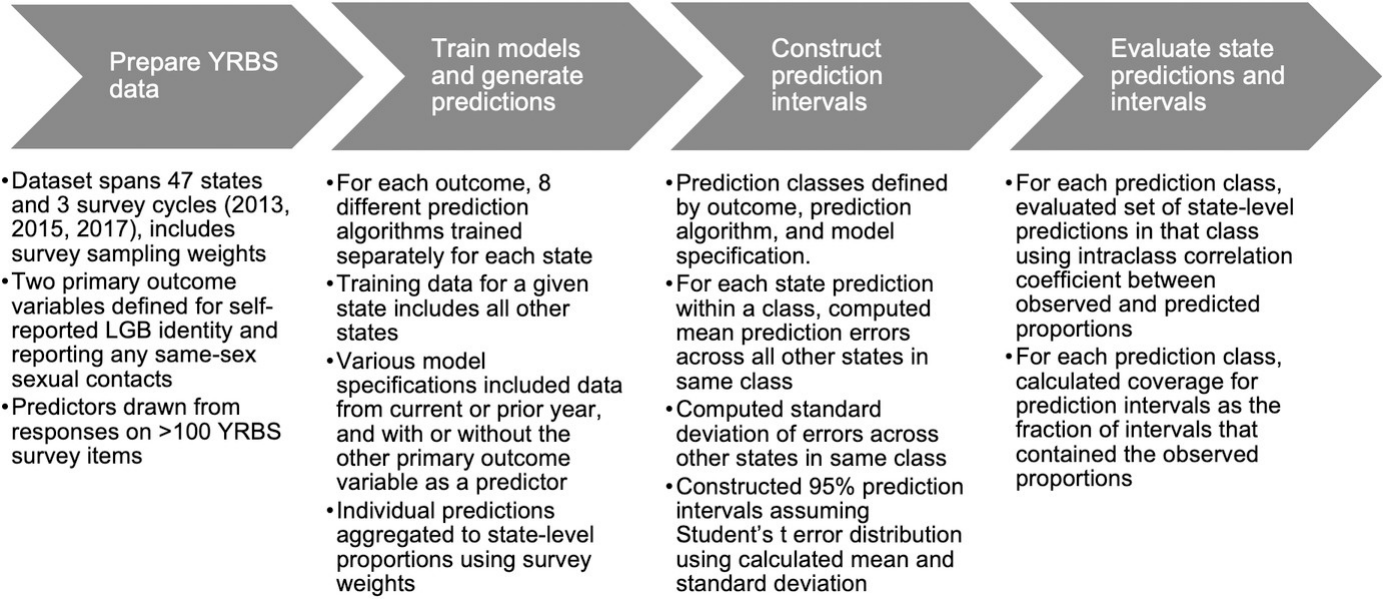
Summary of study approach to training, prediction, and evaluation. Abbreviations: YRBS, Youth Risk Behavior Survey; LGB, lesbian, gay, or bisexual.

The overall aim of the analysis was to develop a predictive model for proportions of high school students reporting LGB identity or reporting any same-sex sexual contacts, in order to impute these proportions in states that did not include these items on their 2017 YRBS questionnaires. We trained separate predictive models for each of the two outcome variables, examining a range of alternative modeling approaches, and we evaluated the predictions from alternative approaches using a leave-one-out cross-validation strategy. Based on model selection criteria, we identified the best-performing model for each of the two primary outcomes, and summarized results as observed versus modeled proportions of high school students reporting LGB identity or same-sex sexual contacts. The following sections elaborate on each of these steps, with a high-level overview depicted in Fig 1.

#### Predicting individual-level YRBS responses

To predict state-level proportions of sexual minority youth, we first trained predictive models for each of the two primary outcome variables at the individual level. We compared the performance of the following predictive modeling approaches: ordinary least squares (OLS), logistic regression, least absolute shrinkage and selection operator (LASSO) penalized linear regression, LASSO penalized logistic regression, ridge penalized linear regression, ridge penalized logistic regression, random forest (RF), and gradient boosted regression trees (GBRT).

Hyperparameter tuning was done to minimize mean squared error in *n*-fold cross validation with each state’s data allocated to only one of the folds such that no state’s data was included in both the training and validation datasets while evaluating hyperparameters. This ensured that we found a set of hyperparameters that performed well when predicting responses in a different state. For the penalized regression models, we tuned the lambda hyperparameter, which penalizes the sum of absolute (LASSO) or squared (ridge) coefficients in the fitted model. A higher penalty will force the model to ignore predictors that do not improve the fit of the model but may also reduce the quality of the predictions if the penalty is too high. An optimal lambda value helps the model pick a subset of predictors that leads to the best final predictors.

For ensemble models (RF and GBRT), we tuned maximum depth, number of trees, learning rate (of the boosted trees), and the number of features to sample for each split. Tuning these hyperparameters helps identify a good trade-off between the quality of predictions from each tree against the quality of the average prediction in the ensemble of trees. For each of the two outcome variables, we compared the performance of models that included the other outcome variable as a predictor against models that did not include the other outcome variable.

#### Generating state-level proportions and prediction intervals

After generating predictions of the two outcome variables for individual YRBS respondents, we created state-level proportion estimates using the survey weights from YRBS. Next, we generated an error distribution around the point estimate for a given state by first calculating the error (residual) of the point estimates for all other states and calculating the mean and standard deviation of these errors. Then, we defined a Student’s t distribution of errors with mean equal to the average residual of the other states, standard deviation equal to the standard deviation of the errors from the other states, and degrees of freedom equal to the number of states represented in the distribution, minus one. This method of defining an error distribution is commonly used to generate prediction intervals for neural networks and performed adequately for our use case based on the coverage of our generated prediction intervals [16].

We generated 95% state prediction intervals with lower bound set at the 2.5^th^ percentile of the error distribution, and upper bound at the 97.5^th^ percentile of the error distribution. We generated separate prediction intervals for three different types of prediction tasks that were used across different subsets of states: (1) prediction based on YRBS data from the same year including the other primary outcome as a predictor, (2) prediction based on YRBS data from the same year without the other outcome variable as a predictor, and (3) prediction based on YRBS data from a previous year without the other outcome variable as a predictor. Thus, a state with 2015 and 2017 YRBS data that included answers to both focal questions would have three prediction intervals for each focal question– one interval for each type of prediction task.

#### Comparative evaluation of predictive models, model selection, and final state-level estimates

We evaluated the predictive models using a leave-one-out cross validation approach at the state level. For each state, we trained the individual-level model on data from all other states, and then used the available data from the held-out state to generate a prediction interval for the primary outcome variable (Fig 1). We repeated this for all the states that had observed data for each outcome. Our evaluation metrics were: (1) the intraclass correlation coefficient (ICC) between our point estimates and those observed in the YRBS data, and (2) prediction interval coverage, expressed as the fraction of observed state proportions contained within our generated prediction intervals.

To generate the final set of state-level estimates, we selected the predictive model for each outcome that had the highest intraclass correlation coefficient. Using the selected model, we predicted survey responses in states in which there were no survey data available for one or both questions. We summarized results as estimated proportions of high school students reporting LGB identity or any same-sex sexual contacts, including both directly observed survey estimates where available and our model-predicted estimates otherwise. All of the code used to generate our predictions is available online [17].

### Ethics statement

The study was reviewed by the Stanford University Institutional Review Board, which determined that the research does not involve human subjects as defined in 45 CFR 46.102(f) or 21 CFR 50.3(g).

## Results

### Model-fitting results

The training dataset included 382,251 responses for the survey item on reported LGB identity, and 320,410 responses for the survey item on sex of sexual contacts. Many states only had training data for one or two years, and the number of questions asked in each state varied (Table 1).

When predicting state-level proportions, the RF and GBRT models had the highest ICCs on both outcome variables, while OLS and logistic regression had the lowest. Penalized linear and logistic models had ICCs that fell between these extremes (Tables 2, 3 and S3, S4 Tables). As expected, ICCs were higher when including the other outcome variable as a predictor. We used the RF over GBRT to predict outcomes in states without outcome data as the RF ICC values were slightly higher in nearly every case.

**Table 2:** Evaluation results from eight algorithms predicting the proportion of students in grades 9–12 reporting lesbian, gay, or bisexual identity in 2017.

	Prediction data		
Model type used to predict individual responses	Same year with other focal question	Same year without other focal question	Previous year without other focal question
OLS	ICC: 0.026 (p-val: 0.472); Coverage: 0.88	ICC: 0.013 (p-val: 0.485); Coverage: 0.9	ICC: 0.068 (p-val: 0.433); Coverage: 0.96
Logistic	ICC: 0.059 (p-val: 0.438); Coverage: 0.92	ICC: 0.013 (p-val: 0.485); Coverage: 0.93	ICC: 0.038 (p-val: 0.463); Coverage: 0.91
LASSO (linear)	ICC: 0.341 (p-val: 0.149); Coverage: 1	ICC: 0.201 (p-val: 0.273); Coverage: 1	ICC: 0.446 (p-val: 0.084); Coverage: 0.91
LASSO (logistic)	ICC: 0.341 (p-val: 0.149); Coverage: 0.96	ICC: 0.196 (p-val: 0.278); Coverage: 0.97	ICC: 0.394 (p-val: 0.121); Coverage: 0.91
Ridge (linear)	ICC: 0.282 (p-val: 0.203); Coverage: 0.96	ICC: 0.141 (p-val: 0.34); Coverage: 0.97	ICC: 0.299 (p-val: 0.202); Coverage: 0.91
Ridge (logistic)	ICC: 0.351 (p-val: 0.14); Coverage: 0.88	ICC: 0.186 (p-val: 0.289); Coverage: 0.93	ICC: 0.352 (p-val: 0.155); Coverage: 0.96
Random forest (linear)	ICC: 0.819 (p-val: 0); Coverage: 0.92	ICC: 0.593 (p-val: 0.008); Coverage: 0.93	ICC: 0.643 (p-val: 0.009); Coverage: 0.91
Gradient boosted regression trees (logistic)	ICC: 0.674 (p-val: 0.003); Coverage: 0.96	ICC: 0.547 (p-val: 0.017); Coverage: 0.97	ICC: 0.587 (p-val: 0.02); Coverage: 0.87

Abbreviations: OLS, ordinary least squares; LASSO, least absolute shrinkage and selection operator; ICC, intraclass correlation coefficient

**Table 3:** Evaluation results from eight algorithms predicting the proportions of students in grades 9–12 reporting any same-sex sexual contacts in 2017.

	Prediction data		
Model type used to predict individual responses	Same year with other focal question	Same year without other focal question	Previous year without other focal question
OLS	ICC: 0.428 (p-val: 0.082); Coverage: 0.96	ICC: 0.372 (p-val: 0.123); Coverage: 0.96	ICC: 0.250 (p-val: 0.264); Coverage: 0.95
Logistic	ICC: 0.585 (p-val: 0.015); Coverage: 0.88	ICC: 0.461 (p-val: 0.062); Coverage: 0.88	ICC: 0.384 (p-val: 0.145); Coverage: 0.95
LASSO (linear)	ICC: 0.843 (p-val: 0); Coverage: 0.96	ICC: 0.733 (p-val: 0.001); Coverage: 0.96	ICC: 0.745 (p-val: 0.002); Coverage: 1
LASSO (logistic)	ICC: 0.835 (p-val: 0); Coverage: 0.92	ICC: 0.687 (p-val: 0.002); Coverage: 1	ICC: 0.750 (p-val: 0.002); Coverage: 1
Ridge (linear)	ICC: 0.721 (p-val: 0.001); Coverage: 0.92	ICC: 0.625 (p-val: 0.008); Coverage: 0.88	ICC: 0.482 (p-val: 0.077); Coverage: 0.95
Ridge (logistic)	ICC: 0.709 (p-val: 0.001); Coverage: 0.88	ICC: 0.589 (p-val: 0.014); Coverage: 0.88	ICC: 0.489 (p-val: 0.072); Coverage: 0.95
Random forest (linear)	ICC: 0.839 (p-val: 0); Coverage: 0.92	ICC: 0.708 (p-val: 0.001); Coverage: 0.96	ICC: 0.725 (p-val: 0.003); Coverage: 0.95
Gradient boosted regression trees (logistic)	ICC: 0.814 (p-val: 0); Coverage: 0.92	ICC: 0.690 (p-val: 0.002); Coverage: 0.92	ICC: 0.726 (p-val: 0.003); Coverage: 1

Abbreviations: OLS, ordinary least squares; LASSO, least absolute shrinkage and selection operator; ICC, intraclass correlation coefficient

S5 Table reports the top 20 predictors across the two full RF models based on variable importance (permutation) scores. S1 Fig shows how the performance of the different prediction algorithms related to the number of predictors available. The predictive performance of the tree-based algorithms surpassed those of the other algorithms as the number of predictors available increased.

For states with observed proportions of respondents reporting LGB identity, the mean absolute error (MAE) of out-of-sample predictions was 0.76 percentage points (pp) when predicting using data from the same year and including the answer to the other focal question (same-sex sexual contacts), 1.05pp when predicting using data from the same year but without the other focal question, and 0.85pp when predicting using data from a previous year without the other focal question. Analogous MAEs for the same-sex sexual contact proportions were 0.61pp, 0.88pp, and 0.77pp, respectively.

Coverage of the out-of-sample prediction intervals for the RF models ranged between 91% and 96% depending on the outcome and prediction dataset. For every state, the observed proportion of students reporting LGB identity was greater than the observed proportion reporting any same-sex sexual contact. We verified that the predictions preserved this relationship.

### State-level estimates of proportions reporting LGB identity or same-sex sexual contacts

**Fig 2:**
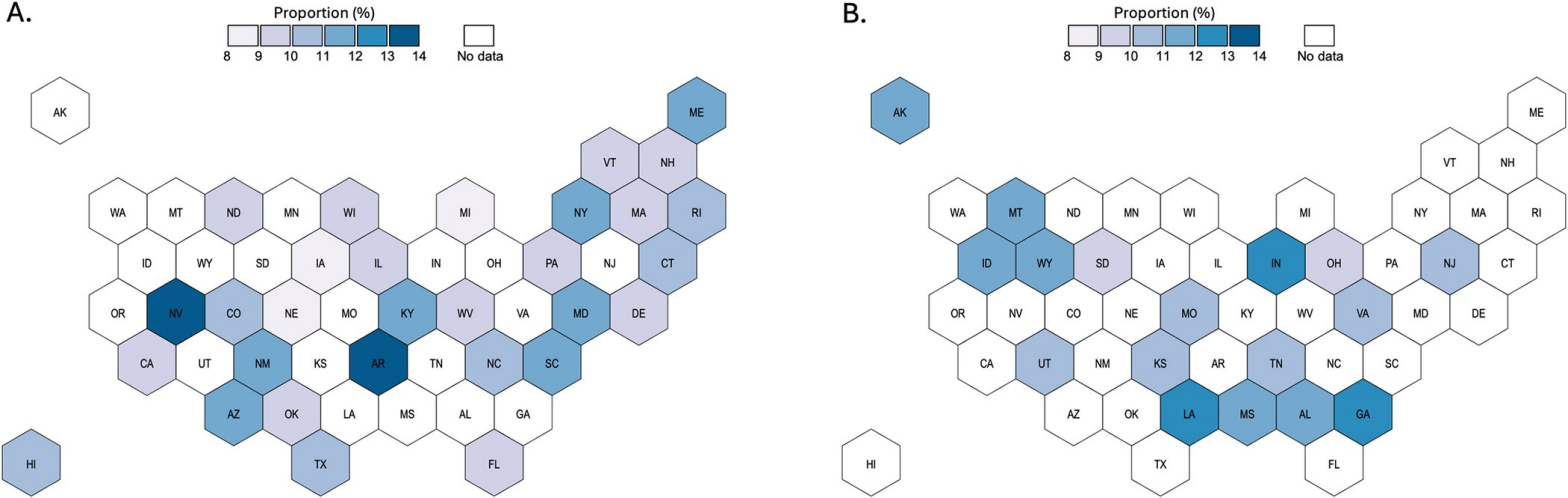
Observed (Panel A) and predicted (Panel B) proportions of students in grades 9–12 reporting lesbian, gay, or bisexual identity in 2017.

**Fig 3:**
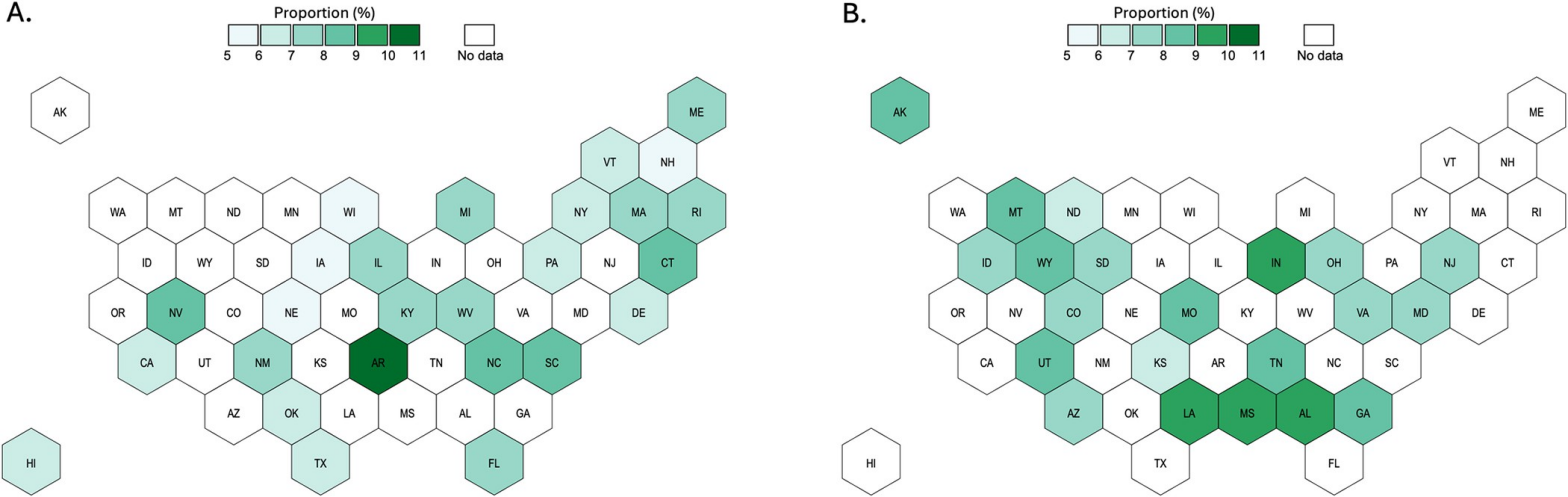
Observed (Panel A) and predicted (Panel B) proportions of students in grades 9–12 reporting any same-sex sexual contacts in 2017.

Figs 2 and 3 show observed and predicted proportions by state using the RF models. S6 and S7 Tables report state-level observed intervals from the YRBS and our prediction intervals. Observed proportions of respondents reporting LGB identity ranged between 8.4% and 13.4%, while the predicted proportions across states without observed proportions, and across different prediction tasks, ranged between 9.4% and 12.9%. Observed proportions of respondents reporting any same-sex sexual contacts ranged between 5.3% and 10.9%, while the predicted proportions where not observed ranged between 7.0% and 10.4%. Results for sex-stratified models of proportions reporting any same-sex sexual contacts are reported in S8 and S9 Tables. Predicted proportions for males where not observed ranged between 3.8% and 8.0%, and for females between 9.7% and 13.3%.

## Discussion

Using a machine learning approach and recent state YRBS data, we generated population estimates of sexual minority adolescents for 47 states. Because of the high salience of these estimates for public health monitoring and program planning, our goal was to supplement data that are available for only a subset of states with predicted estimates for states that do not have direct observations on these measures but do have YRBS data that can inform these predictions.

Comparing across different possible methods for predicting responses to survey items on reported LGB status and sex of sex partners, we found that tree-based ensemble methods commonly used in machine learning applications consistently outperformed traditional linear regression-based models for our prediction tasks. As noted in other areas of research, it is important to benchmark the performance of machine learning models against simpler alternatives [18], and in this application, the additional complexity produced substantial improvements in predictive performance. As in previous studies [6, 7, 19, 20], some of the variables that were most highly correlated with the primary outcome variables were related to suicidal ideation, mental health, substance use, and violence victimization (S5 Table).

Our finding that tree-based ensemble models outperformed other machine learning models is consistent with previous studies focusing on similar binary classification tasks [21, 22]. Analogizing to regression-based approaches, this finding suggests that the relationships between responses to individual YRBS questions and the reported LGB identity and same-sex sexual contacts questions are better modeled as a set of complex non-linear interactions than as a traditional penalized linear model. We found that linear methods designed for high-dimensional data (LASSO, ridge) outperformed the un-penalized OLS and logistic models but did not perform as well as the tree-based ensemble models, suggesting linear penalization is not sufficient to excel at our prediction task.

### Limitations

Our study has several limitations. First, while we modelled responses to items on the YRBS questionnaire, we did not attempt to characterize or adjust for the accuracy of these responses. Previous studies have indicated that willingness to report LGB status may vary systematically across individuals [23]. Second, there are known concerns about the representativeness of surveys relating to sexual minorities [24], which may be exacerbated by non-response at either the item or survey levels. Third, we assumed state-level errors in predicted proportions were distributed identically across states due to our relatively limited numbers of observation units, which may result in mischaracterization of the uncertainty intervals around state-specific estimates in our study.

Another set of limitations derives from the nature of the prediction task. Predicting the size of LGB youth populations from survey data is difficult because reported LGB identity is not highly correlated with the responses to any individual YRBS question. Even when predicting reported LGB identity with the responses to all other YRBS questions and their interactions, no individual was assigned a very high predicted probability of reporting LGB status. Without such strong correlations, prediction will always be difficult, and will result in substantial prediction errors and wide uncertainty intervals, as in our results. It is worth noting, however, that our measurement goal was to develop aggregate-level predictions that may be used by public health decision-makers and not to impute individual-level responses. Among other considerations, this distinction has an important ethical dimension in that public-use research datasets from surveys must meet high standards for protection of privacy and confidentiality of respondents.

We also recognize the complexity of measuring sexual orientation; while we focused on predicting responses to two particular survey measures that operationalize identity-based or behavior-based dimensions of sexual orientation, there is a rich discussion in the literature on the challenges of survey measurement in this domain that is beyond the scope of our study [25, 26]. Finally, we acknowledge that the predicted population sizes of LGB minority youth in this study provide only a starting point for further analyses that can inform public health programs and priorities. An important extension of this work will be to examine possibilities for estimates that are disaggregated below state-level, for example by race and ethnicity, to enable deeper examination of disparities.

## Conclusion

Accurate estimates of LGB population are necessary for informed policy making. Predicted LGB population estimates in this study can raise awareness among state policy makers and the public about what proportion of youth may be exposed to disparate health risks associated with sexual minority status. These estimates support development and implementation of policies that are inclusive and address the health needs of LGB individuals. Understanding the prevalence of sexual minority identities among adolescents can help in advocating for anti-discrimination policies and practices that promote equality and inclusivity, which in turn can have positive effects on the health and well-being of LGB individuals. Estimates of sexual minority youth populations also supply denominators for calculating HIV and STI prevalence and incidence among this group, provide parameter estimates for modeling the impact of various prevention programs, and help public health practitioners develop and implement evidence-based interventions to effectively reduce the burden of infections, likelihood of experiencing violence, and adverse mental health outcomes. Ultimately, the results from this study can help decision makers in public health and education agencies understand the need for community and school interventions to improve the overall health of LGB youth, for example, all-inclusive sex education, positive youth development programs, and gay-straight alliances.

## Supporting information

S1 Table Youth Risk Behavior Survey data availability by state and year. (PDF)

S2 Table Data used to predict sex-stratified proportions of students in grades 9–12 reporting any same-sex sexual contacts in the United States in 2017. (PDF)

S3 Table Evaluation results from eight algorithms predicting the proportions of male students in grades 9–12 reporting any same-sex sexual contacts in 2017. (PDF)

S4 Table Evaluation results from eight algorithms predicting the proportions of female students in grades 9–12 reporting any same-sex sexual contacts in 2017. (PDF)

S5 Table Top 20 predictors based on variable importance scores predicting reported lesbian, gay, or bisexual identity and reporting any same-sex sexual contacts among students in grades 9–12 using YRBS data from 2013–2017. (PDF)

S6 Table Observed and predicted proportions of students in grades 9–12 reporting lesbian, gay, or bisexual identity in 2017, by state and prediction data. (PDF)

S7 Table Observed and predicted proportions of students in grades 9–12 reporting any same-sex sexual contacts in 2017, by state and prediction data. (PDF)

S8 Table Observed and predicted proportions of male students in grades 9–12 reporting any same-sex sexual contacts in 2017, by state and prediction data. (PDF)

S9 Table Observed and predicted proportions of female students in grades 9–12 reporting any same-sex sexual contacts in 2017, by state and prediction data. (PDF)

S1 Fig Prediction loss by number of predictors and prediction algorithm. (PDF)
