# Supplementary material for: State-level population estimates of sexual minority adolescents in the United States: A predictive modeling study
Source: PLoS One. 2024 Jun 27;19(6):e0304175. doi: 10.1371/journal.pone.0304175 (PMC11210845; doi:10.1371/journal.pone.0304175)
Supplement: S1 Table — (PDF) [file pone.0304175.s001.pdf]

Table S1: Youth Risk Behavior Survey data availability by state and year

	YRBS data available, observations (N) and questions (Q)			Data available on reported lesbian, gay, or bisexual Identity			Data available on reporting any same-sex sexual contacts		
State	2017	2015	2013	2017	2015	2013	2017	2015	2013
AK	N:1,332. Q:83	N:1,418. Q:76	N:1,235. Q:71	-	-	-	-	-	-
AL	-	N:1,565. Q:80	N:1,574. Q:76	-	-	-	-	-	-
AR	N:1,682. Q:92	N:2,880. Q:84	N:1,547. Q:79	Y	Y	-	Y	Y	-
AZ	N:2,139. Q:75	N:2,582. Q:68	N:1,623. Q:65	Y	Y	Y	-	-	-
CA	N:1,778. Q:85	N:1,943. Q:81	-	Y	Y	-	Y	Y	-
CO	N:1,493. Q:62	-	-	Y	-	-	-	-	-
CT	N:2,425. Q:30	N:2,398. Q:30	-	Y	Y	-	Y	Y	-
DE	N:2,906. Q:80	N:2,777. Q:82	N:2,756. Q:78	Y	Y	Y	Y	Y	Y
FL	N:6,171. Q:80	N:6,359. Q:76	N:6,089. Q:78	Y	Y	Y	Y	Y	Y
GA	-	-	N:1,992. Q:20	-	-	-	-	-	-
HI	N:6,031. Q:72	N:6,089. Q:68	N:4,631. Q:67	Y	Y	Y	Y	Y	Y
IA	N:1,691. Q:89	-	-	Y	-	-	Y	-	-
ID	N:1,818. Q:87	N:1,760. Q:83	N:1,886. Q:79	-	-	-	-	-	-
IL	N:5,010. Q:88	N:3,282. Q:83	N:3,276. Q:85	Y	Y	Y	Y	Y	Y
IN	-	N:1,912. Q:35	-	-	Y	-	-	Y	-
KS	N:2,413. Q:82	-	N:1,941. Q:71	-	-	-	-	-	-
KY	N:1,997. Q:89	N:2,577. Q:82	N:1,626. Q:75	Y	Y	-	Y	Y	Y
LA	N:1,273. Q:80	-	N:1,107. Q:55	-	-	-	-	-	-
MA	N:3,286. Q:77	N:3,120. Q:73	-	Y	Y	-	Y	Y	-
MD	N:51,087. Q:33	N:55,596. Q:33	-	Y	Y	-	-	Y	-
ME	N:9,501. Q:65	N:9,605. Q:59	N:9,017. Q:57	Y	Y	Y	Y	Y	Y
MI	N:1,626. Q:93	N:4,816. Q:82	N:4,266. Q:79	Y	Y	Y	Y	Y	Y
MN	-	-	-	-	-	-	-	-	-
MO	N:1,864. Q:69	N:1,502. Q:61	N:1,616. Q:56	-	-	-	-	-	-
MS	-	N:2,154. Q:79	N:1,584. Q:74	-	-	-	-	-	-
MT	N:4,741. Q:90	N:4,486. Q:83	N:4,889. Q:83	-	-	-	-	-	-
NC	N:3,151. Q:78	N:6,178. Q:71	N:1,846. Q:66	Y	Y	Y	Y	Y	Y
ND	N:2,142. Q:73	N:2,121. Q:68	N:1,981. Q:63	Y	Y	Y	-	-	-
NE	N:1,427. Q:93	N:1,688. Q:85	N:1,885. Q:78	Y	-	-	Y	-	-
NH	N:12,050. Q:67	N:14,837. Q:56	N:1,634. Q:60	Y	-	Y	Y	-	Y
NJ	-	-	N:1,701. Q:74	-	-	-	-	-	Y
NM	N:5,781. Q:34	N:8,304. Q:32	-	Y	Y	-	Y	Y	-
NV	N:1,667. Q:80	N:1,452. Q:81	N:2,133. Q:75	Y	Y	-	Y	Y	-
NY	N:11,411. Q:63	N:10,834. Q:61	N:10,643. Q:55	Y	Y	-	Y	Y	-

OH	-	-	N:1,455. Q:25	-	-	-	-	-	-
OK	N:1,649. Q:89	N:1,611. Q:81	N:1,474. Q:74	Y	Y	-	Y	Y	-
OR	-	-	-	-	-	-	-	-	-
PA	N:3,761. Q:93	N:2,899. Q:82	-	Y	Y	-	Y	Y	-
RI	N:2,221. Q:77	N:3,462. Q:71	N:2,453. Q:70	Y	Y	Y	Y	Y	Y
SC	N:1,501. Q:90	N:1,358. Q:84	N:1,606. Q:79	Y	-	-	Y	-	-
SD	-	N:1,313. Q:74	N:1,320. Q:69	-	-	-	-	-	-
TN	N:2,043. Q:75	N:4,138. Q:53	N:1,904. Q:79	-	-	-	-	-	-
TX	N:2,113. Q:37	-	-	Y	-	-	Y	-	-
UT	N:1,848. Q:83	-	N:2,195. Q:74	-	-	-	-	-	-
VA	N:3,697. Q:68	N:5,195. Q:72	N:6,935. Q:68	-	-	-	-	-	-
VT	N:20,653. Q:30	N:21,013. Q:48	-	Y	Y	-	Y	Y	-
WA	-	-	-	-	-	-	-	-	-
WI	N:2,067. Q:81	-	N:2,843. Q:69	Y	-	Y	Y	-	Y
WV	N:1,563. Q:89	N:1,622. Q:81	N:1,793. Q:74	Y	Y	-	Y	Y	-
WY	-	N:2,424. Q:81	N:3,015. Q:74	-	Y	-	-	Y	-

Abbreviations: YRBS, Youth Risk Behavior Survey
