# Supplementary material for: State-level population estimates of sexual minority adolescents in the United States: A predictive modeling study
Source: PLoS One. 2024 Jun 27;19(6):e0304175. doi: 10.1371/journal.pone.0304175 (PMC11210845; doi:10.1371/journal.pone.0304175)
Supplement: S2 Table — (PDF) [file pone.0304175.s002.pdf]

Table S2: Data used to predict sex-stratified proportions of students in grades 9-12 reporting any same-sex sexual contacts in the United States in 2017

	Males		Females	
Statistic	Outcome available (training data)	Outcome unavailable (prediction data)	Outcome available (training data)	Outcome unavailable (prediction data)
Number of respondents included	156,405	45,143	160,976	46,420
Number of states included with any YRBS data	30	21	30	21
Number of states included with 2017 YRBS data	26	13	26	13
Number of states included with 2015 YRBS data	23	5	23	5
Number of states included with 2013 YRBS data	12	3	12	3
Number of states also asking the other focal question in 2017 YRBS	26	4	26	4
Unique years of YRBS data included per state, number, mean [min, max]	2.0 [1, 3]	1 [1, 1]	2.0 [1, 3]	1 [1, 1]
Number of questions included per state, median [min, max]	81 [30, 93]	75 [20, 90]	81 [30, 93]	75 [20, 90]
Observed proportion by state per 100k population, mean (SD)	4,478 (906)	-	9,537 (1,293)	-

Abbreviations: LGB, lesbian, gay, or bisexual; YRBS, Youth Risk Behavior Survey; SD, standard deviation
