# Supplementary material for: State-level population estimates of sexual minority adolescents in the United States: A predictive modeling study
Source: PLoS One. 2024 Jun 27;19(6):e0304175. doi: 10.1371/journal.pone.0304175 (PMC11210845; doi:10.1371/journal.pone.0304175)
Supplement: S3 Table — (PDF) [file pone.0304175.s003.pdf]

Table S3: Evaluation results from eight algorithms predicting the proportions of male students in grades 9-12 reporting any same-sex sexual contacts in 2017

	Prediction data		
Model type used to predict individual responses	Same year with other focal question	Same year without other focal question	Previous year without other focal question
OLS	ICC: 0.365 (p-val: 0.128); Coverage: 0.92	ICC: 0.325 (p-val: 0.163); Coverage: 0.92	ICC: 0.163 (p-val: 0.348); Coverage: 0.95
Logistic	ICC: 0.560 (p-val: 0.021); Coverage: 0.92	ICC: 0.422 (p-val: 0.086); Coverage: 0.96	ICC: 0.265 (p-val: 0.25); Coverage: 0.95
LASSO (linear)	ICC: 0.563 (p-val: 0.02); Coverage: 0.96	ICC: 0.417 (p-val: 0.089); Coverage: 0.92	ICC: 0.469 (p-val: 0.084); Coverage: 0.95
LASSO (logistic)	ICC: 0.589 (p-val: 0.014); Coverage: 0.96	ICC: 0.275 (p-val: 0.21); Coverage: 0.92	ICC: 0.424 (p-val: 0.115); Coverage: 0.95
Ridge (linear)	ICC: 0.528 (p-val: 0.031); Coverage: 0.96	ICC: 0.438 (p-val: 0.075); Coverage: 0.96	ICC: 0.383 (p-val: 0.146); Coverage: 0.9
Ridge (logistic)	ICC: 0.620 (p-val: 0.009); Coverage: 0.92	ICC: 0.488 (p-val: 0.048); Coverage: 0.92	ICC: 0.560 (p-val: 0.038); Coverage: 0.95
Random forest (linear)	ICC: 0.673 (p-val: 0.003); Coverage: 0.92	ICC: 0.541 (p-val: 0.027); Coverage: 0.92	ICC: 0.368 (p-val: 0.158); Coverage: 0.9
Gradient boosted regression trees (logistic)	ICC: 0.661 (p-val: 0.004); Coverage: 1	ICC: 0.587 (p-val: 0.014); Coverage: 0.96	ICC: 0.596 (p-val: 0.025); Coverage: 0.95

Abbreviations: OLS, ordinary least squares; LASSO, least absolute shrinkage and selection operator; ICC, intraclass correlation coefficient
