# Supplementary material for: State-level population estimates of sexual minority adolescents in the United States: A predictive modeling study
Source: PLoS One. 2024 Jun 27;19(6):e0304175. doi: 10.1371/journal.pone.0304175 (PMC11210845; doi:10.1371/journal.pone.0304175)
Supplement: S5 Table — (PDF) [file pone.0304175.s005.pdf]

Table S5: Top 20 predictors based on variable importance scores predicting reported lesbian, gay, or bisexual identity and reporting any same-sex sexual contacts among students in grades 9-12 using YRBS data from 2013-2017

Predictor	Question	Lesbian, gay, or bisexual identity model importance. Permutation RMSE/1000 (rank among predictors)	Any same-sex sexual contact model importance. Permutation RMSE/1000 (rank among predictors)
sex	What is your sex? (Female / Male)	21.22 (2)	8.08 (2)
q66	During your life, with whom have you had sexual contact? (Never / Females / Males / Both)	27.94 (1)	
q67	Which of the following best describes you? (Heterosexual / Gay or Lesbian / Bisexual / Not Sure)		14.11 (1)
q64	The last time you had sexual intercourse, did you or your partner use a condom? (Never / Yes / No)	0.67 (13)	4.95 (3)
q26	During the past 12 months, did you ever seriously consider attempting suicide? (Yes / No)	4.25 (3)	1.24 (9)
q65	The last time you had sexual intercourse, what one method did you or your partner use to prevent pregnancy? (8 possible responses)	2.17 (5)	2.91 (6)
q59	Have you ever had sexual intercourse? (Yes / No)	0.63 (15)	3.95 (4)
q25	During the past 12 months, did you ever feel so sad or hopeless almost every day for two weeks or more in a row that you stopped doing some usual activities? (Yes / No)	3.46 (4)	0.96 (11)
stheight	How tall are you without your shoes on?	0.99 (9)	3.26 (5)
q63	Did you drink alcohol or use drugs before you had sexual intercourse the last time? (Never / Yes / No)	0.28 (25)	2.46 (7)
qbullygay	During the past 12 months, have you ever been the victim of teasing or name calling because someone thought you were gay, lesbian, or bisexual? (Yes / No)	1.75 (6)	0.53 (15)
q27	During the past 12 months, did you make a plan about how you would attempt suicide? (Yes / No)	1.70 (7)	0.35 (18)
stweight	How much do you weigh without your shoes on?	0.67 (14)	1.17 (10)
q61	During your life, with how many people have you had sexual intercourse? (0-6+)	0.16 (43)	1.42 (8)
bmipct	Student's BMI percentile (Calculated from stheight and stweight.)	1.03 (8)	0.33 (19)

bmi	Student's BMI (Calculated from stheight and stweight.)	0.85 (10)	0.43 (17)
q19	Have you ever been physically forced to have sexual intercourse when you did not want to? (Yes / No)	0.36 (21)	0.92 (13)
q60	How old were you when you had sexual intercourse for the first time? (Never / 11-17+)	0.39 (19)	0.85 (14)
q62	During the past 3 months, with how many people did you have sexual intercourse? (Never / 0-6+)	0.23 (34)	0.94 (12)
q79	During the past 7 days, on how many days were you physically active for a total of at least 60 minutes per day? (0-7)	0.69 (11)	0.32 (20)

Abbreviations: YRBS, Youth Risk Behavior Survey; RMSE, root mean square error
