# Supplementary material for: State-level population estimates of sexual minority adolescents in the United States: A predictive modeling study
Source: PLoS One. 2024 Jun 27;19(6):e0304175. doi: 10.1371/journal.pone.0304175 (PMC11210845; doi:10.1371/journal.pone.0304175)
Supplement: S6 Table — (PDF) [file pone.0304175.s006.pdf]

Table S6: Observed and predicted proportions of students in grades 9-12 reporting lesbian, gay, or bisexual identity in 2017, by state and prediction data

State	Observed prevalence	Predicted prevalence (data: same year data with other focal Q)	Predicted prevalence (data: same year data without other focal Q)	Predicted prevalence (data: previous year data without other focal Q)
AR	**13.4 (11.9, 15.1)**	14.9 (13.0, 16.7)	15.0 (12.4, 17.5)	13.0 (10.7, 15.3)
AZ	**11.3 (10.1, 12.8)**		9.9 (7.3, 12.4)	12.1 (9.8, 14.3)
CA	**9.3 (8.0, 10.7)**	10.4 (8.5, 12.3)	10.4 (7.9, 13.0)	9.8 (7.5, 12.1)
CO	**10.3 (8.8, 11.9)**		9.3 (6.7, 11.9)	
CT	**10.6 (9.4, 11.9)**	10.2 (8.3, 12.2)	9.6 (7.0, 12.2)	10.4 (8.1, 12.8)
DE	**9.8 (8.8, 10.9)**	9.6 (7.6, 11.5)	10.0 (7.4, 12.7)	9.8 (7.5, 12.1)
FL	**9.9 (9.2, 10.7)**	9.9 (8.0, 11.9)	9.6 (6.9, 12.2)	10.3 (8.0, 12.6)
HI	**10.8 (10.0, 11.6)**	10.9 (9.0, 12.9)	10.7 (8.1, 13.3)	11.3 (9.0, 13.6)
IA	**8.4 (7.2, 9.8)**	9.1 (7.2, 11.1)	9.8 (7.3, 12.4)	
IL	**9.7 (8.9, 10.5)**	10.8 (8.9, 12.7)	10.8 (8.2, 13.4)	10.0 (7.7, 12.3)
KY	**11.1 (9.8, 12.6)**	9.9 (8.0, 11.8)	9.9 (7.4, 12.5)	10.6 (8.3, 12.9)
MA	**9.1 (8.1, 10.1)**	9.9 (8.0, 11.8)	9.3 (6.6, 11.9)	9.6 (7.3, 11.9)
MD	**11.7 (11.5, 12.0)**		9.4 (7.0, 11.9)	9.8 (7.7, 12.0)
ME	**11.3 (10.7, 12.0)**	9.3 (7.5, 11.1)	8.7 (6.3, 11.1)	9.2 (7.1, 11.3)
MI	**8.7 (7.4, 10.2)**	10.3 (8.4, 12.1)	10.6 (8.1, 13.1)	10.5 (8.3, 12.7)
NC	**10.4 (9.4, 11.5)**	11.1 (9.2, 13.1)	11.2 (8.6, 13.8)	11.2 (8.9, 13.5)
ND	**9.2 (8.0, 10.5)**		11.2 (8.7, 13.7)	10.3 (8.0, 12.6)
NE	**8.6 (7.3, 10.2)**	8.7 (6.7, 10.6)	9.2 (6.6, 11.8)	
NH	**9.6 (9.1, 10.2)**	8.5 (6.6, 10.4)	8.6 (6.1, 11.2)	
NM	**11.1 (10.4, 12.0)**	10.6 (8.7, 12.6)	11.3 (8.7, 13.9)	11.4 (9.1, 13.7)
NV	**13.1 (11.5, 14.8)**	11.0 (9.3, 12.8)	10.4 (8.0, 12.8)	10.4 (8.4, 12.3)
NY	**11.2 (10.7, 11.8)**	9.9 (8.0, 11.8)	9.8 (7.2, 12.4)	10.0 (7.8, 12.3)
OK	**9.5 (8.2, 11.0)**	9.1 (7.1, 11.1)	9.9 (7.3, 12.6)	9.7 (7.4, 12.0)
PA	**9.1 (8.2, 10.0)**	9.9 (7.9, 11.8)	10.3 (7.7, 12.8)	9.9 (7.6, 12.2)
RI	**10.9 (9.7, 12.3)**	10.3 (8.4, 12.3)	10.4 (7.8, 13.0)	11.0 (8.7, 13.3)
SC	**11.2 (9.7, 12.9)**	11.3 (9.4, 13.3)	11.0 (8.4, 13.6)	
TX	**10.5 (9.2, 11.9)**	10.8 (8.9, 12.8)	11.8 (9.2, 14.3)	

VT	**10.0 (9.6, 10.4)**	10.0 (8.1, 12.0)	10.0 (7.4, 12.6)	9.2 (6.9, 11.5)
WI	**9.6 (8.4, 10.9)**	9.6 (7.7, 11.6)	10.4 (7.9, 13.0)	
WV	**9.2 (7.9, 10.8)**	9.9 (8.0, 11.9)	10.0 (7.4, 12.6)	10.9 (8.7, 13.1)
AK			**11.5 (8.9, 14.0)**	11.0 (8.7, 13.2)
AL				**11.4 (9.1, 13.7)**
GA				**12.5 (10.2, 14.7)**
ID			**11.2 (8.7, 13.8)**	11.2 (8.9, 13.4)
IN				**12.1 (9.8, 14.3)**
KS			**10.1 (7.5, 12.6)**	9.4 (7.2, 11.7)
LA			**12.9 (10.4, 15.5)**	10.8 (8.5, 13.0)
MO			**10.3 (7.7, 12.8)**	9.7 (7.5, 12.0)
MS				**12.0 (9.7, 14.2)**
MT			**11.1 (8.5, 13.6)**	10.1 (7.8, 12.4)
NJ				**10.7 (8.4, 13.0)**
OH				**9.9 (7.7, 12.2)**
SD				**9.7 (7.4, 11.9)**
TN			**10.6 (8.1, 13.2)**	11.4 (9.1, 13.6)
UT			**11.0 (8.4, 13.6)**	10.4 (8.2, 12.7)
VA			**10.5 (7.9, 13.0)**	10.5 (8.3, 12.8)
WY				**11.1 (8.8, 13.3)**

The bold entries identify the estimates shown for each state in Figure 2. Predictions for states with observed proportions are "out-of-bag" (generated without using data from the state the prediction was made for). All predicted proportions are for 2017.
