# Supplementary material for: State-level population estimates of sexual minority adolescents in the United States: A predictive modeling study
Source: PLoS One. 2024 Jun 27;19(6):e0304175. doi: 10.1371/journal.pone.0304175 (PMC11210845; doi:10.1371/journal.pone.0304175)
Supplement: S7 Table — (PDF) [file pone.0304175.s007.pdf]

Table S7: Observed and predicted proportions of students in grades 9-12 reporting any same-sex sexual contacts in 2017, by state and prediction data

State	Observed Prevalence	Predicted prevalence (data: same year data with other focal Q)	Predicted prevalence (data: same year data without other focal Q)	Predicted prevalence (data: previous year data without other focal Q)
AR	**10.9 (9.5, 12.5)**	10.3 (8.7, 11.9)	10.8 (8.6, 13.0)	10.0 (8.1, 11.9)
CA	**6.8 (5.7, 8.0)**	6.6 (5.0, 8.2)	6.6 (4.4, 8.9)	6.9 (4.9, 8.8)
CT	**8.2 (7.2, 9.4)**	6.1 (4.7, 7.4)	5.6 (3.7, 7.5)	6.1 (4.5, 7.8)
DE	**6.9 (6.1, 7.9)**	7.6 (6.0, 9.1)	7.4 (5.2, 9.6)	7.9 (6.0, 9.8)
FL	**7.6 (6.9, 8.3)**	7.1 (5.5, 8.8)	6.7 (4.5, 8.9)	7.4 (5.5, 9.4)
HI	**7.0 (6.4, 7.7)**	7.4 (5.8, 9.0)	7.4 (5.2, 9.6)	8.0 (6.1, 9.9)
IA	**6.0 (5.0, 7.2)**	7.5 (6.0, 9.0)	7.8 (5.7, 9.9)	
IL	**7.4 (6.7, 8.1)**	7.7 (6.1, 9.3)	7.8 (5.6, 10.0)	7.9 (5.9, 9.8)
KY	**7.2 (6.2, 8.4)**	7.6 (6.0, 9.2)	7.4 (5.2, 9.6)	8.4 (6.5, 10.3)
MA	**7.8 (7.0, 8.8)**	6.2 (4.7, 7.6)	6.2 (4.1, 8.3)	6.8 (4.9, 8.7)
ME	**8.0 (7.5, 8.6)**	7.1 (5.5, 8.7)	6.3 (4.2, 8.4)	6.8 (4.9, 8.7)
MI	**7.4 (6.2, 8.8)**	7.6 (6.0, 9.2)	8.3 (6.1, 10.5)	7.4 (5.5, 9.4)
NC	**8.2 (7.3, 9.2)**	7.5 (5.9, 9.1)	7.6 (5.4, 9.8)	8.1 (6.2, 10.1)
NE	**5.3 (4.3, 6.6)**	6.1 (4.6, 7.7)	6.6 (4.4, 8.7)	
NH	**5.4 (5.0, 5.8)**	6.3 (4.7, 7.9)	6.1 (3.9, 8.3)	
NM	**7.8 (7.2, 8.5)**	7.8 (6.1, 9.4)	7.5 (5.3, 9.7)	7.7 (5.7, 9.6)
NV	**8.7 (7.4, 10.1)**	8.2 (6.6, 9.8)	7.3 (5.1, 9.4)	7.9 (6.0, 9.9)
NY	**6.8 (6.3, 7.3)**	6.3 (4.7, 7.9)	5.7 (3.5, 7.8)	6.1 (4.2, 8.0)
OK	**6.7 (5.6, 8.1)**	7.3 (5.7, 8.9)	7.7 (5.5, 9.9)	7.5 (5.6, 9.4)
PA	**6.2 (5.5, 7.0)**	6.6 (5.0, 8.3)	7.3 (5.2, 9.5)	7.1 (5.2, 9.0)
RI	**7.5 (6.4, 8.6)**	7.4 (5.8, 9.0)	7.3 (5.1, 9.5)	8.2 (6.2, 10.1)
SC	**8.4 (7.1, 9.9)**	8.7 (7.1, 10.3)	8.4 (6.2, 10.7)	
TX	**6.8 (5.8, 7.9)**	7.4 (5.8, 9.0)	7.9 (5.7, 10.1)	
VT	**6.4 (6.1, 6.7)**	6.3 (4.6, 7.9)	5.5 (3.4, 7.7)	5.8 (3.8, 7.7)
WI	**5.7 (4.8, 6.8)**	6.3 (4.7, 7.9)	7.0 (4.8, 9.1)	
WV	**7.2 (6.0, 8.6)**	7.5 (5.9, 9.1)	8.0 (5.8, 10.2)	8.8 (7.0, 10.6)
AK			**8.3 (6.2, 10.5)**	7.8 (5.9, 9.7)

AL				**9.1 (7.2, 11.0)**
AZ		**7.7 (6.2, 9.3)**		10.4 (8.5, 12.3)
CO		**7.2 (5.6, 8.8)**		
GA				**8.3 (6.4, 10.2)**
ID			**8.0 (5.8, 10.2)**	8.0 (6.1, 9.9)
IN				**9.4 (7.5, 11.3)**
KS			**6.9 (4.8, 9.1)**	7.1 (5.2, 9.0)
LA			**10.0 (7.9, 12.2)**	7.5 (5.6, 9.4)
MD		**7.5 (5.9, 9.1)**		8.8 (6.9, 10.7)
MO			**8.2 (6.1, 10.4)**	7.5 (5.6, 9.4)
MS				**9.5 (7.6, 11.4)**
MT			**8.4 (6.3, 10.6)**	8.1 (6.2, 10.0)
ND		**7.0 (5.5, 8.6)**		7.7 (5.8, 9.6)
NJ				**7.8 (5.9, 9.7)**
OH				**7.3 (5.4, 9.2)**
SD				**7.2 (5.3, 9.1)**
TN			**8.3 (6.2, 10.5)**	9.4 (7.5, 11.3)
UT			**8.9 (6.7, 11.1)**	8.7 (6.8, 10.6)
VA			**7.8 (5.6, 9.9)**	8.4 (6.5, 10.3)
WY				**9.0 (7.1, 10.9)**

The bold entries identify the estimates shown for each state in Figure 3. Predictions for states with observed proportions are "out-of-bag" (generated without using data from the state the prediction was made for). All proportion predictions are for 2017.
