# Supplementary material for: State-level population estimates of sexual minority adolescents in the United States: A predictive modeling study
Source: PLoS One. 2024 Jun 27;19(6):e0304175. doi: 10.1371/journal.pone.0304175 (PMC11210845; doi:10.1371/journal.pone.0304175)
Supplement: S8 Table — (PDF) [file pone.0304175.s008.pdf]

Table S8: Observed and predicted proportions of male students in grades 9-12 reporting any same-sex sexual contacts in 2017, by state and prediction data

State	Observed Prevalence	Predicted prevalence (data: same year data with other focal Q)	Predicted prevalence (data: same year data without other focal Q)	Predicted prevalence (data: previous year data without other focal Q)
AR	**5.7 (4.3, 7.5)**	7.7 (5.9, 9.5)	7.6 (5.5, 9.6)	6.5 (4.4, 8.6)
CA	**4.5 (3.3, 6.1)**	4.3 (2.3, 6.3)	4.3 (2.1, 6.5)	3.6 (1.6, 5.7)
CT	**5.7 (4.6, 7.2)**	3.9 (2.0, 5.7)	3.6 (1.6, 5.7)	3.7 (1.9, 5.6)
DE	**4.2 (3.3, 5.4)**	4.0 (2.0, 6.0)	4.0 (1.9, 6.2)	4.6 (2.5, 6.7)
FL	**4.0 (3.3, 4.7)**	4.2 (2.2, 6.2)	4.2 (2.0, 6.4)	4.6 (2.5, 6.7)
HI	**5.2 (4.4, 6.0)**	5.9 (3.9, 7.9)	5.5 (3.3, 7.7)	5.6 (3.5, 7.7)
IA	**3.3 (2.3, 4.7)**	4.4 (2.4, 6.3)	4.6 (2.5, 6.7)	
IL	**4.7 (4.0, 5.7)**	4.8 (2.8, 6.8)	4.7 (2.5, 6.9)	5.3 (3.2, 7.4)
KY	**3.1 (2.2, 4.4)**	4.4 (2.5, 6.4)	4.4 (2.3, 6.5)	4.9 (3.0, 6.8)
MA	**5.0 (4.0, 6.2)**	3.8 (1.8, 5.7)	3.8 (1.7, 6.0)	3.9 (1.9, 6.0)
ME	**5.6 (5.0, 6.3)**	4.1 (2.2, 6.1)	3.6 (1.6, 5.6)	3.6 (1.7, 5.5)
MI	**5.4 (4.0, 7.1)**	5.1 (3.1, 7.1)	4.9 (2.7, 7.1)	4.7 (2.6, 6.8)
NC	**5.1 (4.1, 6.3)**	4.3 (2.3, 6.3)	4.4 (2.2, 6.5)	4.8 (2.7, 6.9)
NE	**2.8 (1.9, 4.3)**	3.7 (1.8, 5.7)	4.0 (1.9, 6.2)	
NH	**3.3 (2.8, 3.7)**	3.6 (1.6, 5.6)	3.5 (1.3, 5.7)	
NM	**5.5 (4.7, 6.4)**	3.9 (2.0, 5.8)	4.0 (1.9, 6.1)	4.0 (2.0, 6.0)
NV	**4.1 (2.9, 5.6)**	3.9 (1.9, 5.9)	4.1 (1.9, 6.3)	4.6 (2.5, 6.7)
NY	**3.9 (3.4, 4.5)**	4.2 (2.3, 6.2)	4.2 (2.0, 6.4)	4.6 (2.5, 6.7)
OK	**2.9 (1.9, 4.3)**	3.3 (1.3, 5.3)	3.7 (1.5, 5.8)	3.9 (1.8, 6.0)
PA	**3.2 (2.5, 4.1)**	4.2 (2.2, 6.1)	4.5 (2.4, 6.7)	4.1 (2.1, 6.2)
RI	**4.8 (3.7, 6.3)**	4.6 (2.6, 6.6)	5.0 (2.9, 7.2)	5.0 (2.9, 7.1)
SC	**7.2 (5.5, 9.4)**	5.5 (3.6, 7.3)	5.1 (3.1, 7.1)	
TX	**4.5 (3.4, 6.0)**	4.6 (2.6, 6.6)	4.6 (2.4, 6.7)	
VT	**3.7 (3.4, 4.1)**	4.7 (2.7, 6.7)	4.5 (2.4, 6.7)	3.9 (1.8, 6.0)
WI	**3.6 (2.6, 4.9)**	4.0 (2.1, 6.0)	4.1 (1.9, 6.3)	
WV	**5.1 (3.7, 6.8)**	4.9 (2.9, 6.8)	5.1 (2.9, 7.2)	5.2 (3.1, 7.3)
AK			**5.1 (3.0, 7.2)**	5.1 (3.0, 7.1)

AL				**5.6 (3.6, 7.7)**
AZ		**4.4 (2.5, 6.4)**		8.0 (5.9, 10.1)
CO		**3.9 (1.9, 5.8)**		
GA				**5.0 (3.0, 7.1)**
ID			**4.1 (1.9, 6.2)**	4.1 (2.1, 6.2)
IN				**6.6 (4.5, 8.6)**
KS			**4.0 (1.9, 6.2)**	4.2 (2.2, 6.3)
LA			**7.8 (5.6, 9.9)**	5.1 (3.0, 7.1)
MD		**4.3 (2.3, 6.3)**		5.3 (3.3, 7.4)
MO			**4.8 (2.7, 7.0)**	4.5 (2.4, 6.5)
MS				**6.7 (4.7, 8.8)**
MT			**4.6 (2.5, 6.7)**	4.6 (2.5, 6.7)
ND		**4.3 (2.4, 6.3)**		5.3 (3.3, 7.4)
NJ				**4.6 (2.5, 6.7)**
OH				**3.8 (1.8, 5.9)**
SD				**4.4 (2.3, 6.5)**
TN			**5.3 (3.1, 7.4)**	5.2 (3.2, 7.3)
UT			**5.5 (3.3, 7.6)**	5.5 (3.4, 7.6)
VA			**4.7 (2.6, 6.9)**	5.4 (3.3, 7.4)
WY				**6.0 (3.9, 8.1)**

The bold entries identify the estimates shown for each state in Figure 3. Predictions for states with observed proportions are "out-of-bag" (generated without using data from the state the prediction was made for). All proportion predictions are for 2017.
