# Supplementary material for: State-level population estimates of sexual minority adolescents in the United States: A predictive modeling study
Source: PLoS One. 2024 Jun 27;19(6):e0304175. doi: 10.1371/journal.pone.0304175 (PMC11210845; doi:10.1371/journal.pone.0304175)
Supplement: S9 Table — (PDF) [file pone.0304175.s009.pdf]

Table S9: Observed and predicted proportions of female students in grades 9-12 reporting any same-sex sexual contacts in 2017, by state and prediction data

State	Observed Prevalence	Predicted prevalence (data: same year data with other focal Q)	Predicted prevalence (data: same year data without other focal Q)	Predicted prevalence (data: previous year data without other focal Q)
AR	**16.5 (14.1, 19.1)**	13.3 (11.0, 15.7)	14.1 (10.7, 17.6)	13.8 (10.6, 16.9)
CA	**9.3 (7.5, 11.4)**	9.1 (6.4, 11.8)	9.4 (5.8, 12.9)	13.8 (10.6, 16.9)
CT	**10.9 (9.2, 12.7)**	8.1 (5.7, 10.6)	7.7 (4.3, 11.0)	10.4 (7.0, 13.7)
DE	**9.7 (8.3, 11.3)**	11.3 (8.7, 13.9)	11.3 (7.8, 14.8)	8.4 (5.2, 11.5)
FL	**11.4 (10.4, 12.6)**	10.3 (7.6, 13.0)	9.4 (5.9, 12.9)	11.2 (7.9, 14.5)
HI	**8.5 (7.5, 9.5)**	8.4 (5.7, 11.1)	8.7 (5.1, 12.3)	10.3 (6.9, 13.7)
IA	**8.4 (6.7, 10.5)**	10.3 (7.7, 12.9)	10.7 (7.2, 14.1)	10.8 (7.6, 14.0)
IL	**10.3 (9.2, 11.6)**	10.9 (8.2, 13.6)	11.1 (7.5, 14.6)	
KY	**10.9 (9.1, 13.0)**	10.5 (7.8, 13.2)	10.3 (6.7, 13.8)	10.3 (6.9, 13.7)
MA	**10.7 (9.3, 12.3)**	8.8 (6.2, 11.4)	8.6 (5.1, 12.0)	11.9 (8.5, 15.3)
ME	**10.7 (9.8, 11.6)**	10.0 (7.3, 12.7)	9.3 (5.7, 12.8)	9.4 (6.0, 12.7)
MI	**9.5 (7.7, 11.7)**	10.0 (7.3, 12.7)	11.6 (8.1, 15.1)	10.1 (6.7, 13.5)
NC	**11.5 (10.0, 13.1)**	11.0 (8.3, 13.7)	10.9 (7.3, 14.4)	10.2 (6.8, 13.6)
NE	**7.8 (6.1, 10.1)**	8.5 (5.8, 11.2)	9.1 (5.6, 12.6)	11.7 (8.3, 15.1)
NH	**7.7 (7.0, 8.4)**	8.7 (6.1, 11.4)	8.4 (4.8, 11.9)	
NM	**10.1 (9.0, 11.2)**	11.5 (8.8, 14.1)	11.0 (7.5, 14.6)	
NV	**13.6 (11.4, 16.2)**	12.7 (10.0, 15.3)	10.9 (7.5, 14.3)	11.3 (7.9, 14.7)
NY	**9.9 (9.1, 10.6)**	8.4 (5.8, 11.0)	7.3 (3.9, 10.8)	11.8 (8.5, 15.1)
OK	**10.9 (8.9, 13.2)**	11.4 (8.7, 14.1)	11.9 (8.3, 15.4)	7.8 (4.5, 11.0)
PA	**9.4 (8.2, 10.8)**	9.4 (6.7, 12.1)	10.3 (6.7, 13.8)	10.8 (7.4, 14.2)
RI	**9.9 (8.3, 11.8)**	10.2 (7.5, 12.9)	9.9 (6.3, 13.4)	10.0 (6.6, 13.4)
SC	**9.5 (7.6, 11.7)**	11.7 (9.1, 14.2)	11.6 (8.1, 15.0)	11.4 (8.1, 14.7)
TX	**9.1 (7.5, 10.9)**	10.3 (7.7, 13.0)	11.2 (7.8, 14.7)	
VT	**9.1 (8.6, 9.7)**	8.8 (6.1, 11.5)	7.2 (3.7, 10.7)	
WI	**8.0 (6.5, 9.8)**	8.9 (6.2, 11.5)	10.1 (6.6, 13.5)	7.8 (4.4, 11.1)
WV	**9.2 (7.4, 11.5)**	9.8 (7.1, 12.5)	10.7 (7.2, 14.2)	
AK			**12.4 (8.9, 15.9)**	12.6 (9.6, 15.6)

AL				**10.6 (7.3, 13.9)**
AZ		**11.1 (8.4, 13.7)**		12.7 (9.4, 16.0)
CO		**10.6 (7.9, 13.2)**		13.3 (10.0, 16.6)
GA				**11.7 (8.4, 15.0)**
ID			**12.2 (8.7, 15.7)**	11.9 (8.6, 15.2)
IN				**12.3 (9.0, 15.6)**
KS			**10.2 (6.8, 13.7)**	10.1 (6.8, 13.4)
LA			**13.0 (9.5, 16.5)**	11.1 (7.8, 14.4)
MD		**10.7 (8.1, 13.3)**		12.2 (8.9, 15.5)
MO			**11.4 (7.9, 14.8)**	10.0 (6.7, 13.3)
MS				**12.6 (9.3, 15.9)**
MT			**12.8 (9.3, 16.3)**	12.1 (8.8, 15.4)
ND		**9.7 (7.0, 12.3)**		10.2 (6.9, 13.5)
NJ				**11.0 (7.7, 14.4)**
OH				**10.9 (7.6, 14.3)**
SD				**10.6 (7.3, 13.9)**
TN			**11.7 (8.3, 15.2)**	13.2 (9.8, 16.5)
UT			**11.6 (8.1, 15.1)**	11.1 (7.8, 14.4)
VA			**10.1 (6.6, 13.6)**	11.5 (8.2, 14.8)
WY				**12.5 (9.2, 15.8)**

The bold entries identify the estimates shown for each state in Figure 3. Predictions for states with observed proportions are “out-of-bag” (generated without using data from the state the prediction was made for). All proportion predictions are for 2017.
