# Supplementary material for: State-level population estimates of sexual minority adolescents in the United States: A predictive modeling study
Source: PLoS One. 2024 Jun 27;19(6):e0304175. doi: 10.1371/journal.pone.0304175 (PMC11210845; doi:10.1371/journal.pone.0304175)
Supplement: S1 Fig — (PDF) [file pone.0304175.s010.pdf]

Figure S1. Prediction loss by number of predictors and prediction algorithm

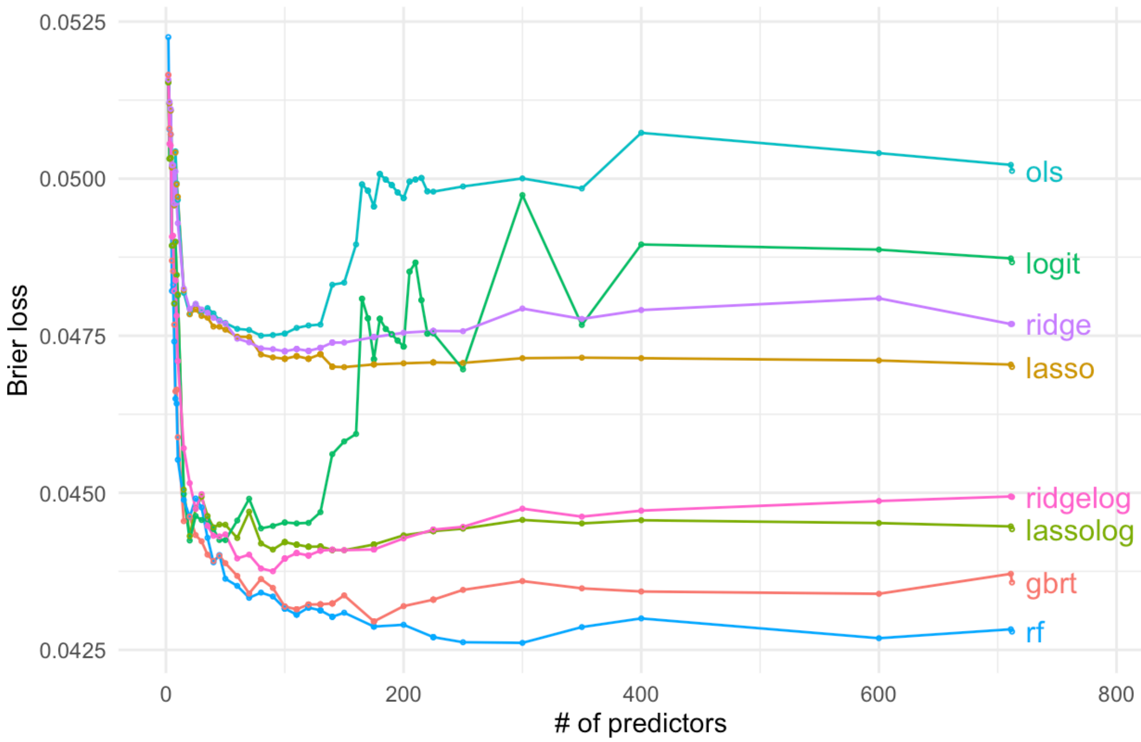

Models were trained to predict 2017 same-sex sexual contact YRBS responses in a held-out dataset. The predictors were selected as the top K (X axis) according to their variable importance based on permutation loss in the training data. The figure clearly shows that the tree-based algorithms outperform the penalized linear models once the number of predictors grows past 50.
